# Complete mitochondrial genome of *Chironomus nipponensis*, new record from China (Diptera: Chironomidae)

**DOI:** 10.1080/23802359.2022.2087564

**Published:** 2022-06-24

**Authors:** Mi Shen, Yufei Li, Yuping Yao, Yue Fu

**Affiliations:** Hubei Key Laboratory of Economic Forest Germplasm Improvement and Resources Comprehensive Utilization, Hubei Collaborative Innovation Center for the Characteristic Resources Exploitation of Dabie Mountains, Hubei Zhongke Research Institute of Industrial Technology, College of Biology and Agricultural Resources, Huanggang Normal University, Huanggang City, Hubei, P. R. China

**Keywords:** Nematocera, *Chironomus*, phylogenetic relationship

## Abstract

The complete mitochondrial genome of *Chironomus nipponensis* Tokunaga, 1936 was sequenced and assembled from the whole genome data. The mitochondrial genome length was 16184 bp and contained 22 transfer RNA genes, 13 protein-coding genes, 2 ribosomal RNA genes, and 1 D-loop control region. Phylogenetic and taxonomic analysis based on the concatenated nucleotide sequences of 37 genes from 14 related species was reconstructed. The phylogeny revealed that *C. nipponensis* is closely related to three other *Chironomus* species, which is consistent with the traditional morphological studies.

*Chironomus nipponensis* Tokunaga, 1936, belonging to the subfamily Chironominae, has been recorded in Japan and South Korea (Tokunaga 1936; Kim et al. 2012). This species was recorded for the first time in China in Henan Province, Xinyang City, which is located in the transition zone of the Palearctic and Oriental regions (Wang et al. 2020).

*Chironomus nipponensis* was collected from Jingangtai Valley (31.7329 N′115.5470E′ alt. 365 m, Shangcheng County, Xinyang City, Henan Province, China) on 18. VIII. 2020 by Yue Fu (email: fuyue20190125@163.com). The specimen was stored at the Biodiversity Herbarium of Huanggang Normal University (http://shengwu.hgnu.edu.cn/2018/1130/c435a7076/page.htm, Yue Fu, fuyue20190125@163.com) under the voucher number HGNU- Ydbs128. Total DNA was extracted using the DNeasy animal tissue kit (QIAGEN, Germany) and sequenced using the Illumina NovaSeq 6000 platform. Raw data were assembled with NOVOPlasty (Dierckxsens et al. 2017) and annotated using the MITOS web server (Bernt et al. 2013). Evolutionary analyses were conducted in PhyloSuite (Zhang et al. 2020) with several plug-in programs. MAFFT (Katoh and Standley 2013) was used for sequence alignment. PartitionFinder2 (Lanfear et al. 2016) was used to select the best-fit partitioning schemes and models. A maximum-likelihood tree (1000 bootstrap replicates) was accessed using IQ-tree (Minh et al. 2013; Nguyen et al. 2015). Tree topology was visualized and edited using iTOL (Letunic and Bork 2019).

The complete mitogenome of *C. nipponensis* was 16,184 bp in length, and included 13 protein-coding genes (PCGs), 22 transfer RNA (tRNA) genes, and 2 ribosomal RNA (rRNA) genes, totaling 37 genes, and 1 control region. The genomic nucleotide composition was A:T:C:G = 39.02%: 37.05%: 14.47%: 9.39%. The total length of the 13 PCGs in the mitochondrial genome was 11,103 bp. Four gene overlapping regions, with a total overlapping length of 17 bp, were observed; the two longest overlapping regions (7 bp) were located between atp8/atp6 and nad4/nad4l. There are 26 intergenic spacers with a total length of 829 bp, with individual lengths ranging from 2 to 504 bp. The longest intergenic space was located between trnE and trnF. The initiation codons of PCGs complied with the ATN rule: there were 4 genes (*nad2, nad3, nad1*, and *nad6*) with ATT as the start codon, 6 genes (*cox2, atp6, cox3, nad4, nad4l*, and *cob*) with ATG as the start codon, 1 gene (atp8) with ATA as the start codon, 1 gene (nad5) with GTG as the start codon, and 1 gene (cox1) with TTG as the start codon. Except for nad4 with TAG as the stop codon, all other PCGs had TAA as the stop codon. The length of the tRNA genes ranged from 66 to 72 bp, with a total length of 1495 bp. The lengths of the 12S rRNA and 16S rRNA were 822 and 1336 bp, respectively.

Phylogenetic analysis was performed on the 37 genes from 14 species using the IQ-tree with the maximum likelihood method, and two taxa were used as an outgroup to reveal the phylogenetic relation (Figure 1). The ML analysis showed species of the same family clustered together. ((*Chironomus nipponensis* + *Chironomus flaviplumus*) + *Chironomus tepperi*) and *Chironomus kiiensis* were clustered together with a 100% bootstrap support value, all belonging to *Chironomus*. The species of *Chironomus* closely related to (*Polypedilum vanderplanki* + *Stenochironomus tobaduodecimus*), belonged to tribe Chironomini, subfamily Chironominae, which is consistent with the traditional morphological classification. The mitochondrial genome of *C. nipponensis* will provide a valuable resource for further taxonomic research and phylogenetic studies of *Chironomus*.

**Figure 1.**
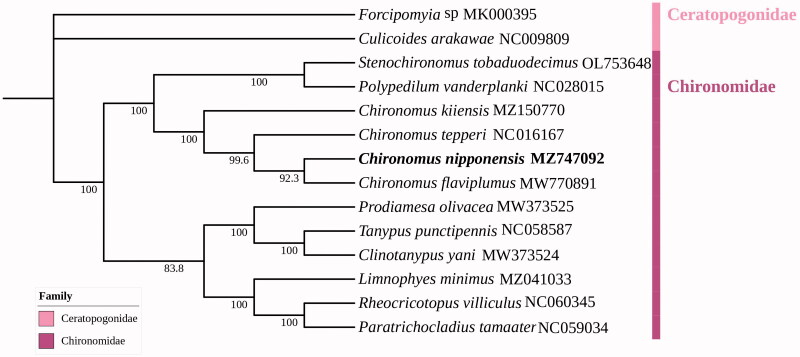
Phylogenetic tree based on 37 genes of the mitogenomes of 12 related species inferred by maximum likelihood method (ML tree). The concatenated sequences of *Forcipomyia* sp. (MK000395) and *Culicoides arakawae* (NC009809) were used as outgroups.

## Ethical approval

This study did not involve any ethical issues.

## Author contributions

Mi Shen designed the model, drafted the paper, and analyzed the data. Yufei Li assembled and annotated the mitochondrial genome. Yuping Yao performed the phylogeny analysis. Yue Fu reviewed the manuscript critically for intellectual content and approved the final version to be published. All authors agree to be accountable for all aspects of the work.

## Data Availability

The data that was newly obtained in this study are available with NCBI under the accession number MZ747092 (https://www.ncbi.nlm.nih.gov/nuccore/MZ747092). The associated BioProject, SRA, and Bio-Sample numbers are PRJNA804390, SRR18578466, and SAMN27163299, respectively.
